# Words matter: Translanguaging in medical communication skills training

**DOI:** 10.1007/s40037-020-00595-z

**Published:** 2020-05-19

**Authors:** Pilar Ortega, Josh Prada

**Affiliations:** 1 Departments of Emergency Medicine and Medical Education, College of Medicine, University of Illinois, Chicago, IL USA; 2 Spanish Applied Linguistics for World Languages and Cultures, Indiana University-Purdue University, Indianapolis, IN USA

**Keywords:** Medical communication skills, Medical Spanish, Medical humanities, Translanguaging, Bilingualism

## Abstract

Medical communication across languages is gaining attention as the multilingual character of local, regional, and national populations across the world continues to grow. Effectively communicating with patients involves not only learning medical terminology, but also understanding the community’s linguistic practices, and gaining the ability to explain health concepts in patient-centered language. Language concordance between physicians and patients improves patient outcomes, but methods to teach communication skills for physicians are usually limited to the majority or official language. For example, in U.S. medical schools increased demand for physician skills in other languages, such as Spanish, has resulted in renewed academic discourse about best practices in teaching practical communication skills for physicians. In language education, translanguaging is an approach that integrates and validates multilingual individuals’ real use of language, which often includes *non-standard* words, regionalisms, and mixed influences from multiple languages, such as Spanglish or Chinglish. Efforts to improve medical language concordance by teaching a second language to medical students would benefit from an understanding of patient-centered communication strategies, such as is supported by translanguaging. Teaching effective communication skills to physicians should evolve and engage with the fluid linguistic attributes of culturally and linguistically diverse patient populations. In this eye opener, we first introduce the translanguaging perspective as an approach that can increase attention to patient-centered communication, which often includes spontaneous practices that transcend the traditional boundaries of named languages, and then present examples of how translanguaging can be implemented in medical education in order to sustainably enhance equity-minded patient-accessible medical communication.

## Introduction

Expressing health-related concepts in ways that transcend or disregard normative language boundaries is common in multilingual, multicultural environments. Take, for example, Spanish-speaking United States (U.S.) patients who may use what are typically perceived as ‘English technical terms’ that have acquired popular usage in the community, such as /em-ar-ái/ for MRI, or /re-fíl/ for medication refill. Relatedly, when giving instructions in Spanish regarding not drinking from a straw when recovering from a tonsillectomy, the doctor may explain the importance of not using a “popote” /po-po-teh/ to her Mexican patient, a “pajilla” /pa-hí-yah/ to her Nicaraguan patient, or a “cañita” /ka-nyí-tah/ to her Peruvian patient. When the post-tonsillectomy patient is a twelve-year-old who is part of a family where the parents prefer (mostly) Spanish for communication but the patient prefers (mostly) English, the doctor tells the patient not to use a “straw” while using the regional Spanish equivalent “pitillo” /pi-tí-yoh/ when explaining these instructions to her Colombian parents. These cases illustrate that effectively communicating with patients involves not only learning accurate monolingual medical terminology, but also gaining an understanding of community linguistic practices and preferences [1]. These emergent (bottom-up) linguistic practices, however, do not typically find a way into language courses for medical purposes, as they are deemed to fall outside of professional discourse. By not recognizing these practices, medical educators may leave out much more than vocabulary items: they ignore the presence and communication needs of local multilingual communities.

Currently, medical communication in languages other than English is gaining attention as the multilingual character of our society continues to grow. For example, in the U.S. context, the Spanish-speaking community is the largest minority language group [2], while in Australia, Chinese speakers are the largest group with 2.5% Mandarin and 1.2% Cantonese speakers [3], and Turkish speakers form the largest immigrant linguistic group in Germany [4]. Reflecting the super-diversity of the Western world, an increasing body of evidence demonstrates that medical language concordance improves patient outcomes and satisfaction [5] and, in places like the U.S., federal law mandates equitable provision of care for patients regardless of national origin or language preference [6]. Furthermore, U.S. medical schools are tasked by the Liaison Committee on Medical Education with teaching physicians to communicate with patients, including core content standards of communication and interpersonal skills and cultural competency [7].

The fluid nature of language generates variability in vocabulary and pronunciation as dialectal, cross-cultural, and cross-linguistic influences [8] operate across levels, even in presumably language concordant encounters (e.g., English-speaking physician and patient) [9]. Even interpreter-mediated encounters, in which a professional interpreter is present throughout the medical interaction and may help to bridge a potential language discordance between physician and patient, the words that are interpreted are only useful insofar as they are truly understood by the patient [9–11]. Considering the above, strategies to teach effective communication skills to physicians and health professionals should evolve and engage with the fluid linguistic attributes of diverse patient populations. It has further been suggested that effectively teaching medical communication skills in diverse languages will require interdisciplinary collaboration among language professionals, such as linguists/language professors, interpreters/translators, and medical experts, such as physicians and medical educators [1, 12, 13]. To do this, we draw on the translanguaging perspective and argue for its inclusion in medical education settings—such as clinical skills training and medical second language courses—in order to sustainably enhance patient-accessible medical communication skills.

## Integration of translanguaging in medical education settings

Simply put, the translanguaging approach in language education emphasizes the integration of multilingual individuals’ use of language, which may incorporate *non-standard* features, regionalisms, and influences from multiple languages [14] and, in doing so, it encourages us to consider language practices that go beyond monolingual vernaculars as linguistic practices in their own right. Whereas medical school educates medical students in the language of medicine, it must also help students to translate complex medical concepts and terminology into words that patients can understand by accounting for variables such as health literacy, cultural norms, and language preferences. Patient-physician communication must be about effectively conveying meaning, an approach that requires re-formulating and often de-jargonizing medical rhetoric in order to focus on “patient-centered language” [15]. In its essence, this re-formulation of medical language is one way in which physicians must routinely translanguage in order to be understood. By including linguistic features that have traditionally been regarded as “broken” and “incompletely acquired,” translanguaging helps validate multilingual speakers—such as patients—and their lived experiences of health and illness, in their own words.

The term translanguaging emerged in the 1990s and refers to the unbound linguistic practices multilingual speakers engage in in their day-to-day interactions, centralizing the multilingual individual’s repertoires. The translanguaging perspective encourages us to understand bi-/multilingual practices from the multilingual individual’s standpoint, not from the monolingual speaker’s [14]. Typical linguistic interactions among multilingual individuals often involve creative, critical and spontaneous practices that transcend the traditional boundaries of named languages (e.g., English, Spanish, Turkish). Minority language speakers, even those who may not self-describe as multilingual, often combine linguistic or cultural influences, such as sporadic words or acronyms from the majority/official language and regional words from specific areas, in their day-to-day interactions and healthcare communications.

In addition to teaching medical communication skills in English, a majority of U.S. medical schools report offering opportunities for medical Spanish education [16], but little is currently known about what such educational programs entail. Courses that focus exclusively on medical Spanish terminology or lack learner clinical skills assessment may not adequately address patient-centered communication skills [1]. Applying the concept of translanguaging to the medical clinical skills or medical Spanish classroom can be achieved through acknowledging how local populations use their linguistic repertoires in the medical context and including such linguistic practices in the curriculum. To that end, medical language curricula should first address the presence of minority groups, particularly the ethnolinguistic minority groups and sizable immigrant groups in the area, and reflect their linguistic (and cultural) practices. For example, instructors might share data with students regarding the minority language speakers in the region, and students may be asked to observe the use of language among these groups of speakers at local public spaces or in their own clinical observations; such first-hand observational encounters may help students to appreciate how the notion of standard language does not capture the linguistic reality of these populations. Students may be asked to complete a reflection in response to open-ended prompts or questions following a clinical encounter to help them in considering the use of standard or hybrid language practices in a particular encounter in the target language.

Similarly, resources used for medical second language education, such as glossaries or sample cases, should address linguistic variation not only to represent those language varieties typically acknowledged as representative of purely monolingual communities, but should also include local linguistic practices that transcend the boundaries of monolingualism and are often ascribed the rank of subpar or hybridized. It is by unsettling the status quo in monolingual representations of medical language that medical language students will develop the skills to meaningfully establish connections with local multilingual (and often oppressed/marginalized) community members. While it may seem challenging to expand the content and terminology of a medical language course to the potentially endless combinations afforded by multilingual communities, it is important to recognize that the goal of a medical language course is not to fully encapsulate and learn all possible medical terms and communication skills for a given community, but rather to increase the provider’s ability to communicate effectively with a linguistic minority population. Understanding real language use among such a vulnerable population is a critical skill in medical language communication skills acquisition.

The elements of didactics, self-study, clinical experiences, and learner assessment (including performance skills assessment such as standardized patient encounters [17]), have been established by expert consensus as necessary components of medical language curricula [1]. Tab. 1 summarizes proposed examples of how translanguaging may be incorporated into these required elements of medical language courses. For example, this approach seizes valuable opportunities to engage community members—such as community health workers, patients, or interpreters—as guests in medical education settings. Medical school curricula require students to participate in core and elective clinical clerkships; faculty should maximize the value of these clinical experiences by aligning medical language courses with clinical exposures at clinical sites that see a large percentage of patients in the target linguistic population. Further, student groups who grew up speaking the “target language” at home or in the community (i.e., heritage speakers) can be engaged (for course credits) as part of classroom role-plays or cultural-linguistic discussions, providing students with a valuable model of how vernacular multilingual repertoires of locals are deployed in real life in the community. University language courses play a crucial role in promoting “what counts” as medical language, and thus, it is fundamental for medical schools to develop alliances with critical language scholars who can help students (and more often than not, faculty members) debunk the notion that the so-called academic language is the only way to convey scientific notions. Therefore, institutions can help keep medical language education up to date with evolving local, regional, national, and global linguistic realities by promoting collaborative educational research and team-teaching between professionals in medicine and those in applied linguistics [18] and by regularly assessing specific outcomes measures [19], including faculty and learner attitudes and ideologies with regards to combining community vernaculars—such as the so called Spanglish, Chinglish and Hinglish, as well as regionalisms of health-related terms—into patient communication.

**Table 1:** Proposed strategies to incorporate translanguaging in medical language education

Medical language curricular element	Translanguaging pedagogical strategy
Didactics	Include data on ethnolinguistic minority groups at the local, regional, and national level
	Invite community members as guest educators (e.g., community health workers, patients, interpreters)
	Increase cross-disciplinary collaboration between medical and applied linguistics professionals in curriculum design and teaching of medical language didactics
Resources for self-study or homework	Use books or glossaries that incorporate not only pure/standard language but also local linguistic practices
	Create (or have students create) supplemental resources that reflect local linguistic practices
	Partner with students who grew up speaking the “target language” at home in a minority(ized) situation (e.g., Turkish in Germany) and have them serve as teaching assistants or provide supplemental practice for enrolled students
Clinical experiences	Assign students to conduct a small ethnographic observation of community language practices and complete a guided reflection
	Provide service-learning experiences at clinical sites with high percentage patients of target linguistic minority population
	Task students with creating an infographic, presentation, or poster using patient-centered language for a local community that incorporates local language usage (e.g., regionalisms)
Learner assessment	Collect data on learner attitudes and ideologies pertaining to language variation in medical settings
	Integrate translanguaging moments in standardized patient or role-play cases that reflect linguistic practices typical of local community members
	Involve community members whose language use is representative of a local variety to play patient roles in evaluated clinical encounters

Medical language education is a complex and evolving field. Inclusion of translanguaging as a pedagogical element in medical language courses is expected to take place gradually. Admittedly, there may be some resistance to the concept of using and teaching “non-standard” language in professional medical language courses, a concept that may be perceived as challenging the normativity of monolingualism and prescriptivist nature of language teaching. However, it is likely that many medical educators have already embraced translanguaging into general clinical skills education without realizing it, since patient-centered communication—not technical language knowledge—is the principal goal of clinical skills education for medical students and is considered to be “the foundation for the safe and effective practice of medicine” [20].

## Conclusion

Given the ever-changing landscape of language, the community itself can serve as a rich source of linguistic knowledge. Medical students, particularly heritage speakers who may have acquired multilingual skills in their homes [8], are themselves members of the community at large prior to becoming medical professionals. Exploring the possibilities of translanguaging in medical education settings can be viewed as an opportunity to engage community members and heritage language speakers in the professional medical community as ambassadors of their day-to-day linguistic practices that can enhance accessible patient-physician communication both in general clinical skills training and in language-specific courses (e.g. medical Spanish). Future studies should evaluate the incorporation of course elements that impact language concordant communication beyond medical terminology alone, such as regionalisms, cultural concepts, and observed communication skills assessment.

The perspective presented herein goes beyond the teaching of (foreign/second) languages for medical purposes; it emphasizes the multilingual speaker’s experience as worthy of inclusion in spaces in which it has traditionally been excluded, such as professional academic discourse [21]. In doing so, translanguaging acknowledges multilingual/multidialectal practices as communicatively full and valid [22]. It serves as a call to action for medical educators, physicians, and trainees to re-evaluate effective strategies in communication with patients, to develop self-awareness of limitations, and to avoid a one-size-fits-all approach. Such equity-minded medical communication training can be applied in any language and health professions school not as a skill to be conquered but as a career-long journey to embrace, as students become skillful communicators beyond field-specific registers. Medical translanguaging reminds us to adjust our words and communication styles to respectfully acknowledge individual patients’ histories and the dynamic natures of language and medicine, and in so doing, encourages us to contribute to a push for the inclusion and recognition of subjects whose discursive practices have been historically left out of professional spaces, and the pedagogical pathways leading to such spaces.
